# Case Report: An Unusual Case of Fasciculoventricular Pathway

**DOI:** 10.3389/fcvm.2022.818275

**Published:** 2022-02-18

**Authors:** Liu Yang, Zhijian Chen, Min Zhang

**Affiliations:** Department of Cardiology, Union Hospital, Tongji Medical College, Huazhong University of Science and Technology, Wuhan, China

**Keywords:** electrophysiology, arrhythmia, ventricular preexcitation, fasciculoventricular pathway, ablation

## Abstract

A 30-year-old man with an ECG demonstrating ventricular preexcitation with a normal PR interval and a QR pattern in lead V1 was evaluated. Electrophysiology studies showed a normal AH interval and a shortened HV interval at sinus rhythm; while the degree of preexcitation (QRS waveform) and HV interval were not affected by multisite or incremental atrial pacing. These findings implied ventricular preexcitation due to a fasciculoventricular pathway (FVP). Moreover, temporarily blocking FVP conduction mechanically resulted in normal HV interval, absence of delta wave, and an rSR pattern in V1, which indicated incomplete right bundle branch block (IRBBB). These findings suggested the coexistence of FVP and IRBBB, which is very rare.

## Introduction

Fasciculoventricular pathways (FVPs) are uncommon preexcitation variants. Of note, a combination of FVPs with bundle branch blocks is barely observed. In this report, we describe a rare case of FVP with the presence of an incomplete right bundle branch block (IRBBB).

## Case Report

**Figure 1:**
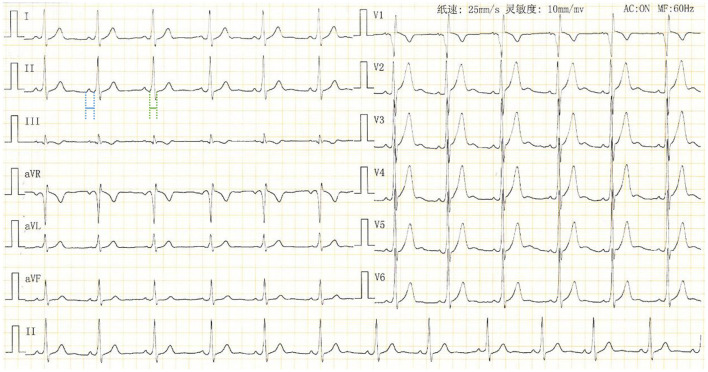
Standard 12-lead surface ECG of the patient upon admission. QRS duration: 102 ms (in green); PR interval: 124 ms (in blue).

A 30-year-old man presented with an aberrant ECG performed 2 years ago. He was asymptomatic with unremarkable medical history. Physical examination findings, laboratory test results, and echocardiographic measurements were within normal range. A 12-lead ECG demonstrated ventricular preexcitation (Figure 1). The patient was admitted to the cardiology unit for consideration of an electrophysiology study (EPS).

The ECG demonstrated the presence of delta waves during sinus rhythm (Figure 1), suggesting a manifest accessory pathway (AP) with antegrade conduction property. Based on the deflection of the delta wave (negative in V1 and positive in lead I and aVL) and very early R-wave progression in the precordial leads (by V2), the ventricular preexcitation is likely mediated by a right septal AP. However, unlike a typical Wolff-Parkinson-White (WPW) pattern, the PR interval of this patient is within normal range with an obvious PR segment (Figure 1). In addition, the QR pattern in V1 is different from what we usually see (a QS or rS pattern) in a patient with ventricular preexcitation conducted over a right septal AP. This necessitates an EPS for accurate diagnosis.

**Figure 2:**
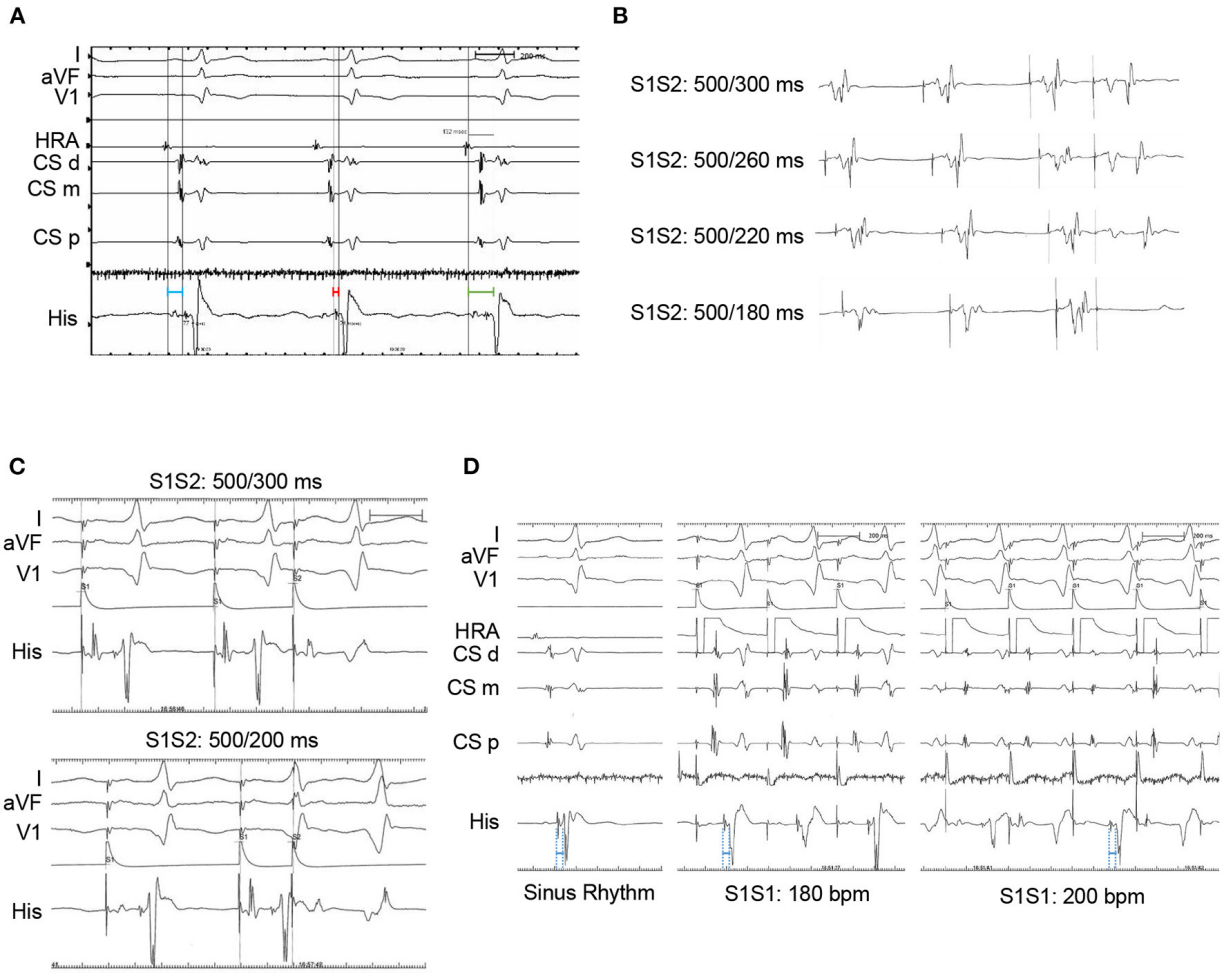
Electrophysiological study. **(A)** Intracardiac electrograms at baseline. PR interval: 130 ms (in green); atrial-His interval: 77 ms (in blue); His-ventricular interval: 24 ms (in red). **(B)** Intracardiac electrograms in His bundle after programed incremental pacing at the right ventricular apex. **(C)** Intracardiac electrograms after programed pacing at right atrium. **(D)** Intracardiac electrograms during sinus rhythm (left panel) or atrial S1S1 stimulation at 180 bpm (middle panel) or 200 bpm (right panel). HV intervals: 24 ms (in blue). HRA, high right atrium; CS, coronary sinus; His, His bundle; d, distal; m, middle; p, proximal; bpm, beats per minute.

**Figure 3:**
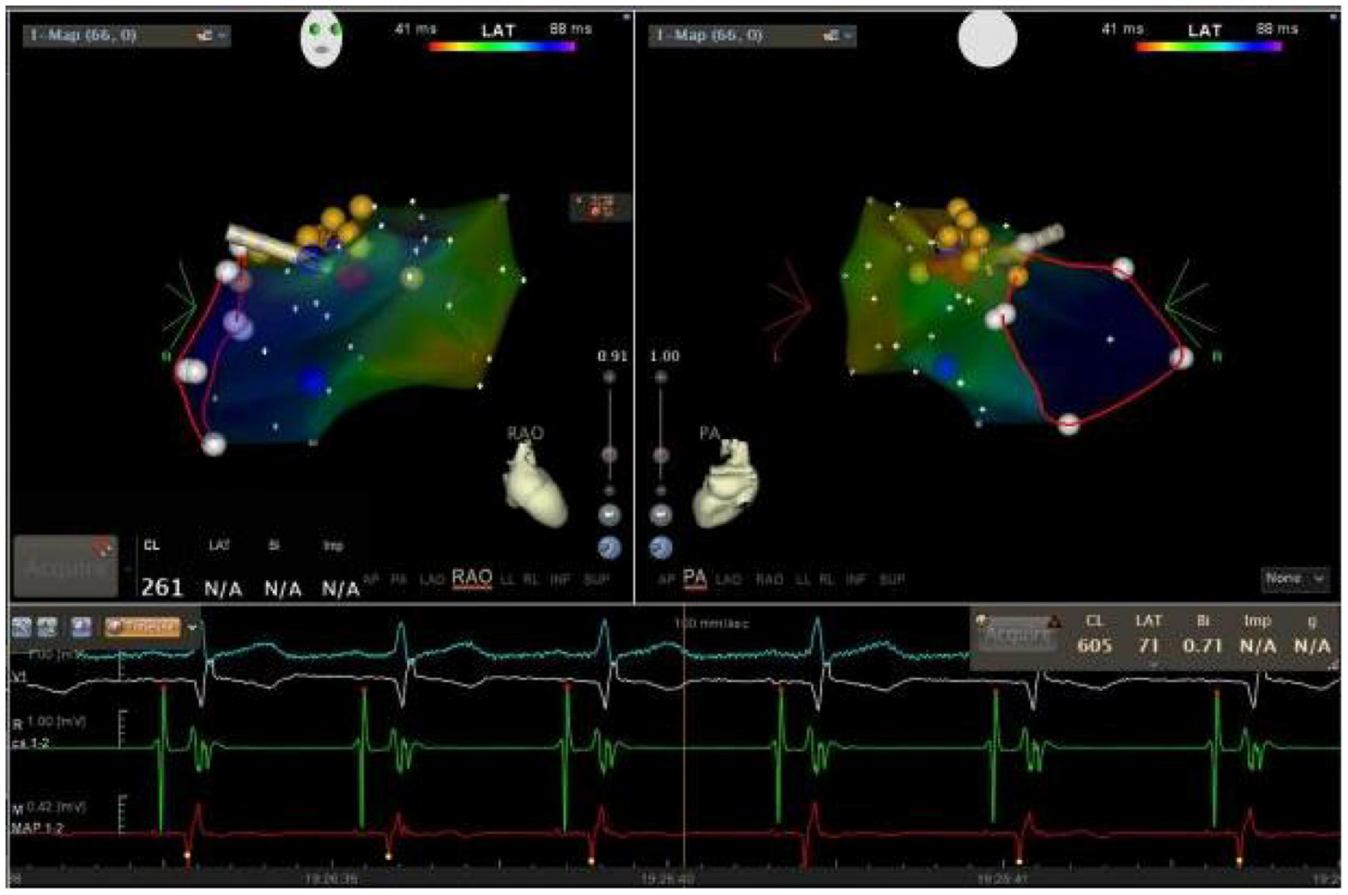
Three-dimensional (3D) mapping of the fasciculoventricular pathway (FVP) using a Carto3 mapping system. The bipolar electrogram of the ablation catheter is shown in red (MAP 1–2). Yellow dots indicate the location where the electrical activity of His bundle was recorded, while the red dots indicate the position where the FVP was mechanically blocked.

**Figure 4:**
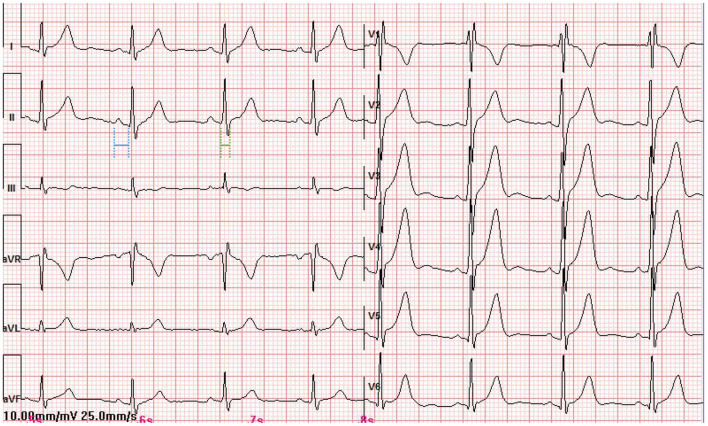
The standard 12-lead surface ECG of the patient when the conductivity of the FVP was temporarily disturbed. QRS duration: 90 ms (in green); PR interval: 135 ms (in blue).

The patient had an EPS performed 3 days later. The baseline intracardiac recording demonstrated normal PR and atrial-His (AH) intervals (130 and 77 ms, respectively) but a shortened His-ventricular (HV) interval (24 ms) (Figure 2A). Right ventricular pacing showed a decremental and concentric ventriculoatrial conduction (Figure 2B). Programed incremental atrial pacing demonstrated a prolonged AV interval (Figure 2C). From sinus rhythm to atrial S1S1 pacing at 180 and 200 bpm, gradually prolonged AH intervals and short but fixed HV intervals at 24 ms were observed (Figure 2D). Notably, the QRS waveform remained unchanged during atrial pacing (Figures 2C,D), or in spontaneous paroxysmal atrial flutter or junctional beats (data not shown). These suggested that the degree of ventricular preexcitation was not affected by the frequency or location of supraventricular stimulation. All these findings favored the diagnosis of fasciculoventricular pathway (FVP) with an anterograde conduction capacity. In addition, three-dimensional (3D) mapping suggested that the earliest ventricular preexcitation occurred in the para-Hisian area (Figure 3). Temporarily blocking FVP conduction mechanically resulted in normal HV interval (Figure 3), absence of delta wave, and an rSR' pattern in V1, which indicated incomplete right bundle branch block (IRBBB) (Figure 4). This explained the QR pattern in V1 in the ECG upon admission. Thus, the final diagnosis of this patient was FVP with IRBBB. No ablation was performed due to the innocent nature of this AP and the non-inducibility of any tachycardia.

## Discussion

The presence of a delta wave with normal PR interval can be seen in the following situations: (1) An AP with a slow antegrade conduction property, such as the Mahaim fiber. The Mahaim fiber is initially identified as a conducting tissue extending from the AV node or His bundle to the ventricular myocardium to form nodoventricular fibers or fasciculoventricular fibers (as it is in this case) (1). Later studies show that Mahaim conduction appears more frequently in atriofascicular pathways, connecting usually the lateral right atrium with the right bundle fascicle (2). It is usually capable of antegrade but not retrograde conduction with a long and decremental conduction property. Preexcitation through the Mahaim fiber displays a small or no delta wave and a normal PR interval, suggesting subtle or non-recognizable ventricular preexcitation. (2) Some left-sided atrioventricular AP. Due to the long distance from the sinus node, conduction to this pathway may be delayed and ventricular preexcitation is not as obvious as APs on the right side. Thus, the PR interval can be within the normal range. (3) AP with delayed intra-atrial conduction. In such a case, the impulse that reaches the AP is delayed and a normal PR interval is displayed.

Fasciculoventricular pathways (FVPs) are the rarest variant of preexcitation (1.2–5.1% in all preexcitation cases) (3, 4), connecting His bundle or bundle branches to the ventricular septum. They are characterized by the presence of a small delta wave, with a normal AH interval and a shortened HV interval at sinus rhythm; while the degree of preexcitation is not affected by multisite or incremental atrial pacing. The main differential diagnosis is anteroseptal AP with rapid conduction property. The criteria that favor an FVP over an anteroseptal AP are: (1) a shorter QRS duration (120 vs. 140 ms); (2) a not-so-short PR interval; (3) a flat or negative delta wave in V1; (4) a narrow delta/R wave in V2; (5) S wave amplitude <20 mm in V1; (6) notching in the descending limb of the S wave in V1; and (7) prolongation of PR interval without changes in the delta wave during intravenous adenosine infusion testing (5). Another differential diagnosis is FVPs that are directly connected from the proximal RBB to the septal ventricle. Mechanically induced proximal RBBB can “correct” aberrant electrical activities (presence of delta wave and shortened HV interval) caused by FVP. However, proximal RBBB is usually more complete (duration of QRS wave > 120 ms) and the basal QR pattern in V1 as well as S waves at the end of QRS complexes in leads I, aVL, V5, and V6 should not be observed prior to EPS. Thus, the concomitant presence of FVPs and IRBBB is more reasonable. FVPs are frequently accompanied by other tachycardia, but they play no active role in producing re-entrant circuits (6, 7). Thus, ablation for FVP is usually not necessary.

To the best of our knowledge, this is the first report demonstrating the coexistence of FVP with IRBBB. In surface ECG, the QR pattern in V1 serves as a hint for the existence of IRBBB. The electrophysiological study is the golden standard for diagnosis. Although the IRBBB, in this case, does not affect the therapeutic strategy for this patient, it broadens our understanding of the co-existence of FVPs with other aberrant electrical activities in the heart.

## Conclusion

Fasciculoventricular pathways (FVPs) are the rarest variant of preexcitation, connecting His bundle or bundle branches to the ventricular septum. A careful evaluation of surface ECGs and EPS findings will help to recognize these pathways. Although radiofrequency ablation is usually not needed for FVPs, a combination of FVPs with other aberrant electrical activities should be noted before a clinical decision is made.

## Data Availability Statement

The original contributions presented in the study are included in the article/supplementary material, further inquiries can be directed to the corresponding author.

## Ethics Statement

Written informed consent was obtained from the individual(s) for the publication of any potentially identifiable images or data included in this article.

## Author Contributions

MZ conceptualized the study. LY, ZC, and MZ collected all the clinical data. LY and MZ wrote the manuscript. All authors contributed to the article and approved the submitted version.

## Conflict of Interest

The authors declare that the research was conducted in the absence of any commercial or financial relationships that could be construed as a potential conflict of interest.

## Publisher's Note

All claims expressed in this article are solely those of the authors and do not necessarily represent those of their affiliated organizations, or those of the publisher, the editors and the reviewers. Any product that may be evaluated in this article, or claim that may be made by its manufacturer, is not guaranteed or endorsed by the publisher.

## References

[1] Gallagher JJ Smith WM Kasell JH Benson Jr. DW, Sterba R, Grant AO. Role of Mahaim fibers in cardiac arrhythmias in man. Circulation. (1981) 64:176–89. 10.1161/01.CIR.64.1.176 7237717

[2] Balaji S Tchou P Kanter R. Mahaim fibers: should they be renamed? Heart Rhythm. (2020) 17:161–2. 10.1016/j.hrthm.2019.07.025 31351139

[3] Sternick EB Rodriguez LM Gerken LM Wellens HJ. Electrocardiogram in patients with fasciculoventricular pathways: a comparative study with anteroseptal and midseptal accessory pathways. Heart Rhythm. (2005) 2:1–6. 10.1016/j.hrthm.2004.10.009 15851255

[4] Suzuki T Nakamura Y Yoshida S Yoshida Y Shintaku H. Differentiating fasciculoventricular pathway from Wolff-Parkinson-White syndrome by electrocardiography. Heart Rhythm. (2014) 11:686–90. 10.1016/j.hrthm.2013.11.018 24252285

[5] de Alencar Neto JN Ramalho de. Moraes SR, Back Sternick E, Wellens HJJ. Atypical bypass tracts: can they be recognized during sinus rhythm? Europace. (2019) 21:208–18. 10.1093/europace/euy079 29788238

[6] O'Leary ET Dewitt ES Mah DY Gauvreau K Walsh EP Bezzerides VJ. Differentiation of fasciculoventricular fibers from anteroseptal accessory pathways using the surface electrocardiogram. Heart Rhythm. (2019) 16:1072–9. 10.1016/j.hrthm.2019.02.011 30763786

[7] Kim YG Nam GB Cho MS. Impact of fasciculoventricular bypass tracts on the diagnosis and treatment of concomitant arrhythmias and cardiac diseases. J Electrocardiol. (2019) 55:34–40. 10.1016/j.jelectrocard.2019.04.002 31078106

